# Challenges of 3D printing technology for manufacturing biomedical products: A case study of Malaysian manufacturing firms

**DOI:** 10.1016/j.heliyon.2020.e03734

**Published:** 2020-04-12

**Authors:** N. Shahrubudin, P. Koshy, J. Alipal, M.H.A. Kadir, T.C. Lee

**Affiliations:** a Department of Production and Operation Management, Faculty of Technology Management and Business, Universiti Tun Hussein Onn Malaysia (UTHM), Parit Raja, 86400, Batu Pahat, Johor, Malaysia; b School of Materials Science and Engineering, UNSW, Sydney, NSW 2052, Australia; c Faculty of Engineering Technology, Universiti Tun Hussein Onn Malaysia (UTHM), Educational Hub Malaysia Pagoh, 84600 Panchor, Johor, Malaysia

**Keywords:** Business, Biomedical products, Additive manufacturing, 3D printing technology

## Abstract

Additive manufacturing has attracted increasing attention worldwide, especially in the healthcare, biomedical, aerospace, and construction industries. In Malaysia, insufficient acceptance of this technology by local industries has resulted in a call for government and local practitioners to promulgate the development of this technology for various industries, particularly for biomedical products. The current study intends to frame the challenges endured by biomedical industries who use 3D printing technology for their manufacturing processes. Qualitative methods, particularly in-depth interviews, were used to identify the challenges faced by manufacturing firms when producing 3D printed biomedical products. This work was able to identify twelve key challenges when deploying additive manufacturing in biomedical products and these include issues related to binder selection, poor mechanical properties, low-dimensional accuracy, high levels of powder agglomeration, nozzle size, distribution size, limited choice of materials, texture and colour, lifespan of materials, customization of fit and design, layer height, and, lastly, build-failure. Furthermore, there also are six challenges in the management of manufacturing biomedical products using 3D printing technology, and these include staff re-education, product pricing, limited guidelines, cyber-security issues, marketing, and patents and copyright. This study discusses the reality faced by 3D printing players when producing biomedical products in Malaysia, and presents a primary reference for practitioners in other developing countries.

## 1 Introduction

Additive manufacturing (AM), also known as 3D printing involves use of digital CAD modelling to build 3D objects by joining materials layer-by-layer [1]. The future demand for this technology lies in its capability to perform different print functions and "print-it-all" structures. These functions are progressively perceived as the driving force for researchers and practitioners even though 3D printing technology has seen significant developments in the last three decades [2]. Moreover, this technology has widely been applied towards the agricultural, biomedical, automotive, and aerospace industries [3]. 3D printing technology has emerged in recent years as a flexible and powerful technique in advanced manufacturing. According to Garcia *et al.* [4], this technology is used widely in the manufacturing industry and medical education field. The different methodologies used for additive manufacturing in the industry include fused deposition modelling (FDM), stereolithography (SLA), selective laser sintering (SLS), and bioprinting [5].

Although the 3D printing technology in Malaysia is clearly in the early developmental stage, this technology is expected to expand and become one of the country's major innovation, particularly in engineering, manufacturing, arts, education, and medicine. The vast majority of researchers have focused exclusively on engineering applications with focus on materials [6], processes [7], techniques [8], and machinery [9] used in optimization. To date, only limited studies have focused on the management aspects of technology, with discussions on the challenges [10] and supply chain management [11]. The existing studies on 3D printing technology have centred on developments in Europe and the USA, with limited focus on biomedical product fabrication, especially in developing countries like Malaysia [12].

Sandstrom [13] was concerned about the adaptation of 3D printing technology in the hearing aid manufacturing industry but the operational and technological challenges faced by producers were neglected. According to Shirazi *et al.* [14], 3D printed biomedical products differ from other printed products because they involve biocompatible materials and clinical testing (*in vitro* and *in vivo*) resulting in operational and technological challenges that are specific to the materials used. Thus, this study discusses the practices involved in manufacturing printed 3D biomaterial products, and, subsequently, fills the gap in the existing research from a management perspective. This study indicates a framework specific to the development of biomedical products. An in-depth interview with three local companies was carried out as the proposed framework to derive real perspectives from real players involved in 3D printing technology for producing biomedical products in Malaysia.

## 2 Literature review

### 2.1 3D printing technology

3D printing can create physical objects from a geometric representation by successive additions of materials [15]. The 3D printing technology has experienced phenomenal development in recent years ever since it was first commercialized in 1980 [16]. Since then, this technology has been principally used to create complex walls [17], endodontic guides [18], sport shoes [19], engine parts for the aviation industry [20], and tumour reconstruction [21]. Commonly, the 3D printing manufacturing process begins with a CAD drawing, followed by objects being sliced into layers, and, lastly, a layer-by-layer 3D build is printed. The 3D printing technology is equipped to fabricate functional parts with a wide range and combination of materials, including aluminium alloy [22], thermoplastic filaments [23], zirconia [24], carbon fibre-reinforced polymer composites [25], hydrogels [26], nanogels [26], and others. An ideal 3D printed biomaterial should morphologically mimic living tissue, be biocompatible, and be easily printable with tuneable degradation rates [27].

There are several types of 3D printing technologies with different functionalities. According to ASTM Standard F2792 [1], this technology can be classified into seven groups: binder jetting, directed energy deposition, material extrusion, material jetting, powder bed fusion, sheet lamination, and vat photo-polymerisation. More than 350 types of industrial 3D printing machines and 450 materials have been identified in the marketplace [28]. These machinery have their own specific applications, and pros and cons. According to Jammalamadaka and Tappa [29], well-known printers for biomedical products are those that are inkjet-based and extrusion-based.

There have been various types of 3D printers used dating back to 1984 with Charles Hull's ideas about a computer system based on stereolithography that uses the STL file format to interpret data in a CAD file [30]. The instructions in the STL file are encapsulated with information, such as the colour, texture, and thickness of the object to be printed [31]. Moreover, different types of printer are designed to print different products, of various scales in various industries, such as healthcare [32], food [33], automotive [34], and architecture [35]. In the 21^st^ century, 3D printing technology began expanding into aircraft manufacturing (producing robotic components), and, subsequently, established the Industry 4.0 paradigm in institutions of higher learning and manufacturing sectors [36]. The following are several advantages derived from using 3D printing technology [37]:

• Customise desired products in a short time;
• Create complex objects and shapes that otherwise might be impossible to create through any conventional method;
• Produce biocompatible products, such as organs or replacement tissues, in a short time compared to conventional methods;
• Cost-effective; and
• Non-requirement of storage of goods or materials.

To sum up, there are several characteristic features for each 3D printing technology application and this could the larger-scale implementation of this technology.

### 2.2 Application of 3D printing for producing biomedical products

Recently, 3D printing technology has rapidly flourished in the industry for the purpose of designing, developing, and manufacturing new products. There are numerous applications of 3D printing technology for producing biomedical products such as drugs, artificial skin, bone cartilage, tissue, and organs, and in cancer research and education.

#### 2.2.1 Drug delivery

In August 2015, the FDA endorsed the use of 3D printing technology for pharmaceutical research and manufacturing [38]. A higher production volume of medicines is achievable through 3D printing technology due to the printer's ability to control the exact drop size and shape. This process allows for higher reproducibility of medicine and formulates a ready dose-shape based on a complex medication discharge profile. An example in drug delivery is the oral tablet produced by 3D printing technology. Oral tablets are the most difficult to manufacture and its successful production by using 3D printing technology is open to further scrutiny [39]. The previous process produces an oral tablet via a complex layer of mixing, milling and dry and wet granulation of powdered ingredients formed through moulds or the compression. Each of these traditional steps involve difficulties, such as drug degradation, form change, and potential problems with formulation or batch failures [34]. Some of the examples of oral tablets are flat-faced [40], spritam (levetiracetam) [41], and paracetamol [42]. Presently, analysts use vapour-stream as a 3D printing method to keep drug measurements on an assortment of surfaces that incorporate dissolvable Listerine tabs [43]. In the meantime, 3D printing technology can also produce antibiotic and chemotherapeutic drugs that are customized according to patient anatomy and clinical presentation [44].

#### 2.2.2 Skin

A process to create a generic 3D-skin structure with minimal costs using 3D printing technology has been successfully achieved. This 3D printed skin is useful as a medium to test pharmaceutical products, beautifying agents, and synthetic items. New 3D human skin models could replace animal trials to assess dermal sensitivity to a medical design. Subsequently, it will enable specialists to achieve precise results after repetitive printing trials [45]. So far, *in vitro* and *in situ* are two existing approaches in skin bioprinting. Both approaches have a similar process except for tissue maturation and the site of printing. The *in vitro* bio-printed skin maturation begins in a bioreactor before it is grafted on the skin. Meanwhile, the *in situ* bioprinting constitutes the direct printing of pre-cultured cells over an injured site. This process supports a recovering wound upon local maturation [46]. Several types of bioprinting technology have been used to prepare 3D-skin, such as laser-assisted [47], micro-extrusion [48], and inkjet bioprinting [49]. To facilitate the 3D printed skin process, a range of natural biomaterials like cellulose [47], alginate [50], GelMA-collagen [51], hydrogels [52], keratinocytes (KCs) [48], fibroblasts (FBs) [48], carbon nanotubes [53], and others have been employed. The availability of suitable biomaterials and technology advancement has resulted in bioprinting being used successfully to fabricate 3D-skin [47].

#### 2.2.3 Bone cartilage

Bone cartilage is a a highly diverse and dynamic tissue, both in function and structure. These properties are due to its ability to perform a wide array of functions, including response to a variety of physical, metabolic, and endocrine stimuli. For mutual injuries, bone has a self-healing capability to form scar-free tissue. However, there are injuries that might emerge in non-union or union delays that require bone regeneration [54]. In this case, 3D printing technology can print tissues to fill out voids in bone defects that are caused by tumour resection, trauma, injury, or infection [55]. This treatment is distinct and provides an alternative to auto-unions and allografts to maintain health or enhance the *in vivo* capacity. Examples of products manufactured by 3D printing technology include cranial portions, bone frameworks, embedding bearings in skull, and bio-fired inserts [56]. Recently, Liu *et al.* [57], suggested that these 3D printing technologies have a higher possibility of repairing fractured bone structure. Meanwhile, Du *et al.* [58], constructed a bioinspired multilayer osteochondral scaffold consisting of hydroxyapatite (HA)/polycaprolactone (PCL) and PCL microspheres using the SLS process. The derived scaffolds present excellent biocompatibility and can induce articulate cartilage formation in cases of osteochondral defects in a rabbit.

#### 2.2.4 Tissues

In a similar manner, 3D printing technology can be utilized to supplant, re-establish, maintain, or enhance the capacity of tissues. The substitute tissues created by 3D printing technology have organized interconnected pores, are biocompatible, and possess excellent mechanical properties. The organized interconnected pores are crucial for wastes removal and improving oxygen and nutrient supply, while the mechanical properties help to match the tissue at the site of the implantation [59]. For example, tissue processes that utilize 3D printing technology have printed some delicate tissue structures such as tooth-supporting tissues and jawbones [60].

#### 2.2.5 Organs

By using 3D printing technology, autologous organs can be printed without any need for immunosuppressive medication or waiting for a donor. This can potentially put an end to the illegal trade in human organs [61]. With the help of 3D printing technology, it is possible to directly print human organs for replacing damaged organs caused by infections, mishaps, or congenital defects [62]. The most commonly printed organs with this technology are the liver, heart valve, ear, and spinal columns [63]. There are currently new ventures to deliver bio-printed organs that are made with the vascular design of a natural organ produced through bio-printing design. The uniform cells can be isolated, cultured *in vitro* and differentiated into specific cell types, which then regenerate specific tissues [64]. According to Jang *et al.* [65], the organ transplantation process is preceded by hydrogel composite systems, and this can be carried out via use of repaired and bio-printed organs in a bioreactor [39].

#### 2.2.6 Cancer research

3D printing technology can revolutionize cancer treatment by printing personalized hydrogels, prostheses, and therapeutic implants [66]. Early diagnosis is essential for reducing cancer mortality and effectively treating the disease. Therefore, the development of accurate and sensitive methods to detect cancer at its early stages has been intensively studied [67]. Thus, utilizing 3D printing technology allows patients to obtain more dependable and accurate information. Presently, 3D demonstration of *in vitro* diseases allows more noteworthy cell feasibility, higher expansion rate, and higher chemo-resistance to anti-cancer medications and helps in providing data related to the qualities of a genuine tumour [68, 69]. For example, 3D printing technology can produce the mandible template using PLA polymer filament or titanium. The template is sterilized according to the Sterrad (low-temperature hydrogen peroxide gas plasma technology) process, which uses H_2_O_2_ plasma and UV irradiation before it is available for treating cancer patients [69].

#### 2.2.7 Educational

**Figure 1:**
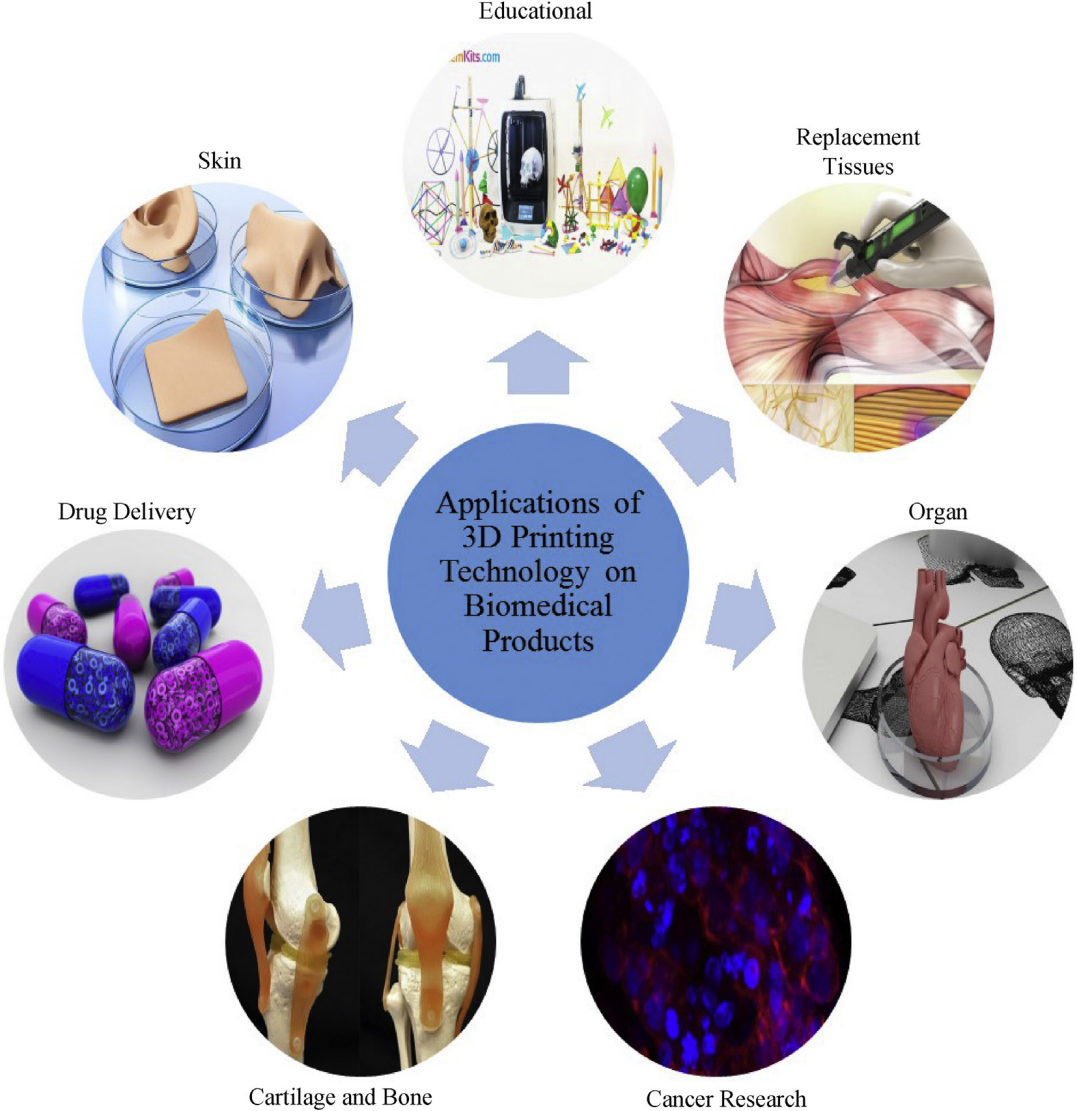
The applications of 3D printing technology for biomedical products.

3D printout models can be used in the learning process to help neurosurgeons hone surgical skills. By implementing 3D printing technology, neurosurgeons can enhance their precision and provide short opportunities to mentor the process throughout the clinical system. As the 3D display provides a re-enactment of a genuine patient's condition, the printer helps the neurosurgeon by providing hands-on experience. Additionally, 3D printing provides visual instrumentation that allows the specialist to share data with patients. Neurosurgeons can share their expertise in pathology and its related concerns to provide long-term care-overview for the immediate prescription of medication to patients [70]. At the same time, 3D printed models can also be used to educate patients and help them to better understand their conditions [71]. Figure 1 shows the present application of 3D printing technology in biomedical products.

## 3 Research framework

This study has developed a conceptual framework related to the challenges faced by 3D printing technology based on previous studies. These challenges are laid out in the following sections.

### 3.1 Processing

#### 3.1.1 Materials

One of the challenges when making bone tissue using 3D printing technology is binder fitting [56]. Not all binders are suitable for use in the sintering process. For example, when producing bone tissue using stereolithography (SLA), only photopolymers are suitable. Among the different binders, organic ones are considered to be the best in producing high quality 3D printed parts or products. However, during the long operational process, this organic binder affects the plastic parts of 3D printing machines [56]. Conversely, Bogue [59] claimed that the selection of a suitable binder is the main challenge when fabricating 3D scaffolds.

Bose *et al.* [56], mentioned that the focus in fabricating bone tissue lies in optimising the mechanical properties of the porous scaffold. This scaffold is generally a ceramic material which is known to have high porosity and low mechanical properties. This challenge was also supported by Egan *et al.* [72], who claimed that engineered scaffold tissue is difficult to optimize due to the complexity involved in interfacing mechanical properties and biological systems. The design requires consideration on mechanical properties, biological performances, and fabrication constraints. The mechanical integrity of the scaffold structure is essential for the promotion of cellular growth. Vasireddi & Basu [73] stated that achieving sufficient mechanical strength and manufacturing feasibility are among the salient challenges. The well-perceived requirement of materials used for fabrication is still inadequate to print varying structures owing to the fact that these aspects include consideration of geometric selection criteria, thickness of the layer, and the minimum ratio between distribution ratios of pore sizes.

Furthermore, the particle size of the powder influences the thickness of the printed layer [73]. Distribution of sizes and shapes of the powder also affect the quality of scaffolds [73]. Lack of pore interconnectivity affects the mechanical properties of 3D scaffolds. The powder must be biocompatible and biodegradable as scaffolds need to promote tissue regeneration after implantation [74]. Hydrogel materials can further aid cell migration and growth to improve the speed of tissue regeneration and repair by replacing a functional material with bionic characteristics resembling extracellular matrix with highly networked 3D structures.

According to Boetker *et al.* [75], the challenge of adopting 3D printing technology is to determine suitable materials that can match the flow properties and requirements for adjusting the nozzle temperature and speed of 3D printing. The flow properties are sensitive to the number of undissolved particles used in the printing process. To date, materials used for 3D printing have been limited by the particle properties. Lee *et al.* [76], mentioned that the challenge for 3D printing technology when producing membranes and membrane module components is the selection of materials for printing [76]. The limited choice of materials suitable for designing membrane modules is the main challenge when producing 3D printed objects. Yap *et al.* [77], suggested that the challenge in printing 3D objects is the limited choice of materials, such as biocompatible or bioresorbable materials. The materials used to print 3D objects are selected based on the printing resolution, 3D printing process, and the material requirements based on similarity and suitability for organs and tissues. The materials must be selected and refined according to the purpose or application in the model [77].

Yap *et al.* [77], found that the challenge in fabricating ophthalmic models includes the texture and colour of products that need to be similar to the printed organ. Meanwhile, according to Chang [78], a challenge faced when producing 3D printed biomedical products is the similarity in colour to the printed product. Multi-extruder 3D printers are available but provide unrealistic results because the melted plastic cools down as soon as it touches the supporting bed and becomes solidified. These multi-extruder 3D printers cannot mix solidified droplets to obtain a continuous full-colour object as the droplets are too large. Some colour 3D printers also try to mix coloured materials before extruding them, but it is difficult to mix melted thermoplastic since it melts >200 °C and cools rapidly if not insulated.

#### 3.1.2 Printers

Pires *et al.* [79], reported that the challenge of 3D printing technology in tissue engineering is with maintaining the accurate dimensions, particularly with the thickness. The accuracy of 3D prints depends on the design of the products as 3D printing technology is not suitable for unsupported long-thin features or flat surfaces. Accuracy will also reduce the size of the part. Scott [80] also mentioned that dimensional accuracy is an issue with fused deposition modelling (FDM)-based printing. However, by using inkjet or poly-jet models, it is possible to obtain very high levels of accuracy and resolution. The dimensional accuracy of a part is determined by several factors, such as the software, XY resolution, screw movements of the machines on the platform and the firmware controls on the projector.

Powder agglomeration is a challenge faced by most manufacturers when producing samples [79]. Larger pore agglomeration results in a non-homogeneous microstructure that eventually eliminates the binder, specifically during sintering which leads to poor densification. Powder morphology and sintering temperature also affects the HA densification, behaviour, microstructure, porosity, and stability. According to Shirazi *et al.* [14], the increasing speed of the laser scanner causes parts of the sample to be solidified. This effect is due to the expanding interactions between the powder and the laser beam over time, which reduces the delivery rate of energy onto the powder bed. However, a laser scanner with a lower speed results in high amounts of energy being transferred to the material, leading to high levels of sintering and in turn, less porosity.

According to Husain *et al.* [81], one of the challenges of current 3D printing technology for producing biomedical products is the difficulty in achieving a nanoscale resolution for clinically relevant biomedical products. Advanced 3D bioprinting techniques were developed to fabricate the next generation of complex biocompatible and biomimetic tissue constructs, such as vascular grafts, dermal dressing, osteochondral tissues, and neural tissues. This statement was also supported by Vasireddi & Basu [73], who said that the challenge of producing 3D scaffolds is the limited resolution of a 3D printer caused by the size of the nozzle [73]. This statement was supported by Yan *et al.* [74], who claimed that problems, such as limited printing resolution during the process, need to be resolved.

### 3.2 Management

From the management's perspective, several challenges were identified. Firstly, according to Sandstrom [13], the challenge related to the adaptation of 3D printed hearing aids in the industry is the re-education of staff to adopt the new technology. The use of software and printers requires the acquisition of new skills by all technicians. Highly skilled technicians are needed in the manual stages involved in producing a 3D hearing aid which include the sculpting, moulding, and curing stages. The technician requires manual and visual skills. Meanwhile, Lind *et al.* [82], claimed that current workers require specific skills in organizing 3D printing technology, especially for biomedical products. The company requires highly skilled workers when implementing 3D printing technology for biomedical products.

The next challenge in adopting 3D printing technology is the cost [13]. The application of 3D printing technology for biomedical products is affected by several cost factors, such as cost of materials, utility, and technological maintenance. In addition, the implementation of 3D printing is associated with various forms of related investment, including hardware, software, and system integration [83].

According to Mellor *et al.* [12], the challenge to develop new businesses in the 3D printing manufacturing industry is related to the size of the company. Proven theories in large enterprises might not be suitable for small businesses. The structure of the organization is a key factor in implementing a 3D printing business. Companies that adopt the technology without redesigning their organizational structure and processes will be the first to encounter difficulties. On the other hand, there are challenges when using 3D printing technology in a different manufacturing industry. It is more feasible to use 3D printing technology in small scale production, especially when there is uncertainty with regard to the demand [84].

Meanwhile, Gao *et al.* [85], found that the challenge of producing a 3D product is the lack of guidelines for a fundamental design. The materials and machines used vary according to the type of biomedical products that need to be produced. Therefore, a designer is required to carry out a trial and error process to obtain the desired products. This claim was also supported by Pavlovich *et al.* [86], who stated that the coordinated standards and regulatory pathways for biomedical products are still lacking. The quality control system should be integrated into the manufacturing process to ensure that the 3D printed biomedical products are well defined, characterized, and meet the regulatory standards.

Lastly, according to Sturm *et al.* [87], using 3D printing technology presents opportunities for cyber-attacks to impact the physical world. This is because 3D printing technology needs internet connectivity to function and is often connected to internal networks. This allows for useful features, such as remote diagnosis troubleshooting, which also opens up the potential for a cyber-attack that compromises the systems remotely [87]. Hoffman and Volpe [88] mentioned that 3D printing technology offers numerous attack surfaces for cyber operations, including the CAD model, the STL file, the tool-path file, and the physical machine itself. As a result, the confidentiality, integrity, availability of data and even fabricated physical components in these systems are at risk. A hacker might target the confidentiality of the digital build files to steal intellectual property and production information or compromise the integrity of the critical data and software to disrupt or sabotage the 3D printing process [88].

**Figure 2:**
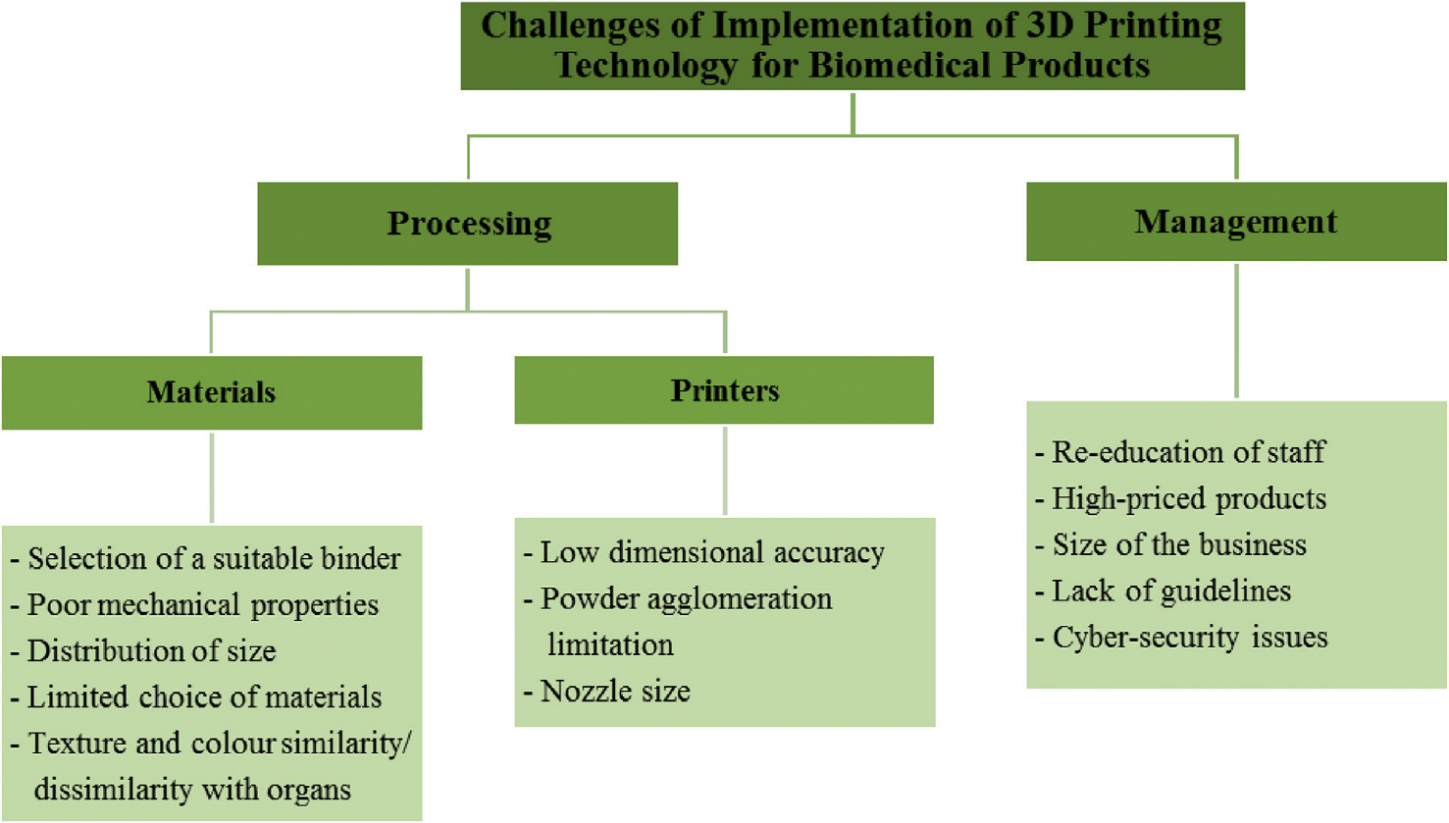
Summary of the challenges of 3D printing technology for biomedical products.

There are various examples of challenges that occur before, during, or after utilizing 3D printing technology for manufacturing biomedical products. Figure 2 shows a summary of the challenges faced when utilizing 3D printing technology to manufacture biomedical products based on the literature.

## 4 Research methodology

This study used a descriptive method to investigate the challenges of utilizing 3D printing on biomedical products in Malaysia, which involved interviews at Company X, Y, and Z. Three persons representing the top management of Companies X, Y, and Z were interviewed. They included an application engineer, a mechanical engineer, and a technical development manager. The qualitative case study method was chosen in this study, as it enables a strong description to address the research questions [89].

### 4.1 Company background

Company X (Respondent 1) was founded in 1990. The company's objective was to develop new uses of 3D printing that have excellent potential. Since its founding, it has gained much experience in solving problems related to software, engineering, and 3D printing services that, together form the backbone of the industry. Furthermore, its open and flexible platforms enable various industries, such as healthcare, automotive, aerospace, art and design, and consumer goods to build innovative applications of 3D printing. This company has become the largest group of software developers in the industry and is one of the largest facilities involved in 3D printing technology in the world. Ultimately, this company is giving its customers a choice of transforming and adopting new digital manufacturing processes and to launch innovations. By utilizing 3D printing, the relevant stakeholders have the potential to change the culture of their industry in the future.

Company Y (Respondent 2) was established in the 2000s with the aim of delivering solutions using the latest high-end technologies. Since then, this company has gained much experience in all aspects of 3D printing technology, especially in 3D bioprinters and has developed customised scientific setups and fabrication machines with programmed system controls. Company Y is also proficient in the field of customized machinery, design museum gallery, rapid prototyping, and professional laser cutting, and highly proficient in 3D living tissue printing.

Since 1980, Company Z (Respondent 3) is the leading and most established CAD, CAM, and CAE solutions provider in Malaysia. This company has grown into a leading product design and manufacturing solutions provider in Malaysia. With numerous clients from corporations, government sectors, and educational partners, this company has established a strong presence locally and expanded its business coverage nationwide. With innovation as its core, this organization effortlessly pursues the best-in-class design solutions and new technologies. In Malaysia, this organization works with Stratasys and is responsible for distributing, selling, and supporting their products in this country. Company Z has branches throughout the country in Penang, Johor, Selangor, and Sarawak. All the branches of the company are capable of distributing CAD/CAM/CAE software solutions using rapid prototyping 3D printers or 3D scanners, and provide consultancy and engineering services, technical support or training, and certification courses to their customers.

## 5 Results and discussion

### 5.1 Challenges faced during processing

#### 5.1.1 Materials

Several challenges were identified after the interview sessions, with a primary focus on the selection of suitable binders, which vary according to the types of products.

Respondent R1 stated that:

*“One of the most challenging tasks faced by engineers and designers is to select a suitable binder for the 3D printing process because each product or design has its own unique binder*.” –R1

Meanwhile, Respondent R2 stated:

*“So, (like for) anything, to produce biomedical products, we have to select suitable binders because not all types of binders are biocompatible.” –*R2

Overall, based on the data collected, Respondents R1 and R2 noted that the selection of suitable binders varied according to the type of product that they wanted to produce. Different binders can have different effects on the biocompatibility of 3D printed biomedical products.

Binder selection is based on the targeted application and capabilities of the printer and printer head. For biodegradable parts, the binder must also be biodegradable, non-toxic, easy to handle, and readily available. In the context of renewable materials, the binder should also be based on natural or renewable resources. Hence, due to these requirements, common binders for 3D-printing are not acceptable [90]. The 3D printing technology might use metal, polymer, hydrogels, resin, glass, ceramic, or polymer as materials to build 3D printed products. The binder is placed layer-by-layer onto a powder by the 3D printing machine's head. Therefore, the selection of suitable binders can be considered a challenge in the utilization of 3D printing technology for biomedical products.

The mechanical strength of products is another challenge in 3D printing technology. In terms of mechanical strength, the challenge of producing 3D printed biomedical products is to determine the suitable strength of 3D printed products. Most engineers worry if the biomedical product is not strong enough and has low mechanical strength. The engineer needs to check the 3D printed biomedical product to determine whether it has adequate tensile strength and stiffness to avoid end products that are of low quality and have low mechanical strength.

According to Respondent R1,

*“You need to check the implant, either if it is suitable or appropriate with the tensile stress strength, Young's modulus or not. All these must (be) measure(d). This is because, the products sometime do not achieve or meet the required mechanical properties. For example, 3D printed biomedical products become brittle and/or have low Young's modulus. So, we can see that the mechanical strength of a product is considered as one of the challenges to produce 3D printed biomedical products.”* –R1

In addition, Respondent R3 stated:

*“For specific 3D printed materials, the mechanical performance of the final print is very important. There are challenges to produce 3D printed biomedical products with good mechanical strength and suitable to the human body. The final printed part mainly depends on the inter-diffusion and re-entanglement between the deposition rasters of the fused polymer.”* – R3

Hence, the development of prostheses is something that is external to the body and often requires the use of materials that not only look like human skin but also matches the strength of the human body part. The well-perceived requirement for material fabrication is still inadequate for various structures because these aspects include criteria, such as geometric selection, layer thickness, and the minimum ratio between pore sizes [73]. Zhang *et al.* [91], found that mechanical properties are sensitive to printing parameters, such as laser scanning speed, powder layer thickness and laser power. Mechanical properties are very important for load-bearing bone tissue reconstruction. Implants with too much stiffness would bear the most stress under pressure, but bone tissue cannot be stimulated by stress. The ideal bone tissue engineering scaffold has macro-pores of ~300–900 μm and a porosity of 60–95%. 3D printing technology, such as SLM, can produce precise porous titanium implants with a pore size of 400–1000 μm, which exhibit excellent osteointegration performance *in vivo* [91]. Furthermore, according to Ji *et al.*, (2018), a scaffold pore diameter ranging between 200 and 400 μm is considered adequate [92]. Meanwhile, Qing *et al.* [93], pointed out that emphasis should be on capsule and tendon reconstruction. The joint capsule and load points of muscle and tendons are unstable and break down due to massive bone defects. Therefore, before processing the implants, the prosthesis is wrapped in polypropylene monofilament knitted mesh (PMKM) [93]. Therefore, the mechanical strength of products is another challenge affecting the utilisation of 3D printing technology.

The size distribution and shape of the powder are challenges in the production of 3D printed biomedical products. Good powder density, including flowability, directly affects the potential to produce good layers during the printing process. Respondent R1 stated that the physical and chemical properties of the powder not only impact the 3D printing process but also affect the properties and quality of 3D printed biomedical products. For example, to produce the trachea, the particle size of the powder must exhibit good biocompatibility and biodegradability, and the trachea must integrate with human tissue to promote tissue regeneration after implantation. Respondent R3 also supported this statement.

Respondent R3 stated that:

*“The size distribution and shape of the powder can affect the optical and thermal properties of particles. The layer properties of the powder largely depend on the powder flowability and density. Lack of pore interconnectivity network caused by the lower bounds of porosity affects the mechanical properties of 3D printed biomedical products”* –R3

From the transcribed data, Respondents R1 and R3 stated that the size distribution and shape of the powder is the main challenge when utilizing 3D printing technology for biomedical products. This is because the size distribution and shape of the powder can directly affect the production of good 3D printed products.

According to Mostafaie *et al.* [94], small powder particles produce a large quantity of small pores distributed throughout the entire part, while large powder particles produce a small number of large pores heterogeneously distributed in the product [94]. Generally, spherical particles within a narrow size range are preferred as they flow more easily and can be deposited more homogeneously. On the other hand, if the size range is too narrow, the powder packing density decreases, which then generates voids and inhomogeneities in the final component. Oversized particles might cause defects in the powder's thin layer and in as the structure of the finished component [95]. Therefore, the selection of the type of powder of an appropriate size and shape is very important for all the companies.

Hence, the limited choice of materials that possess excellent properties for the human body or organ is a challenge faced when producing 3D printed biomedical products. The materials used to produce 3D printed biomedical products must be similar and suitable to human organs and tissues. Respondent R1 stated that materials must be selected properly and scrutinised according to the purpose and application. This is because, sometimes, the customer would request a flexible material that is difficult to break, so the respondent must make it clear how to produce a flexible product that is difficult to fracture. Respondent R2 also supported a similar statement by Respondent R1.

Respondent R1 mentioned that:

“*So, actually we have to examine numerous properties about the material. First, it must be biocompatible to make sure this material can be used in the patient's body*.”

Respondent R1 also stated that:

*“Sometimes the challenge is to choose (the) right material properties. Because sometimes the customer says he wants (a) flexible material (that) does not break. However, this is difficult for us to (achieve). We have to make it clear, how to make it flexible, but not broken or torn. So, (to obtain the) ideal material properties according to customer requirements in materials selection is the challenge for us.”*

Based on the transcribed data, all the respondents stated that the limited choice of materials is the main challenge when utilizing 3D printing technology for biomedical products. This is because the materials must be biocompatible, of good quality, and safe for use in the patient's body.

Therefore, each material has its own properties, which may have varying suitabilities for producing biomedical products using 3D printing technology. There are certain materials that have good printing properties but weak cell-culture properties. It is very challenging to ensure that the material can dissolve in the patient's body and allow it to function naturally. According to Jammalamadaka & Tappa [29], biomaterials are classified based on numerous criteria, such as chemical and physical composition, biodegradability, type of origin, and generation of modifications. The choice of biomaterial is determined depending on the target tissue. Furthermore, Gopinathan & Noh [96] pointed out that the biomaterial properties include the printability, biocompatibility, cytocompatibility, and bioactivity of the cells after printing. Therefore, the selection of appropriate materials is very important for all companies. According to the requirements of the desired tissue and organ, the biomaterials should be selected and can be modified to regenerate the appropriate tissue structure or organ.

Furthermore, in 3D printing, products, texture, and colour play a huge role in making the products stand out. According to Respondent R3, customers request products that are identical to the true organ so that they want to feel like they are really doing an emergency surgery. The old machines allow printing with different materials but with limited colour choice. Therefore, they invented new 3D printing machines so that coloured products can be printed.

Respondent R3 stated:

*“When the customers perform the operation or surgical training, (the) colour of the 3D organs is white. (However), (a) 3D organ or 3D part must have colour. So, they request (that we) make (the) products similar to a true organ.”* – R3

Hence, Respondent R3 stated that the texture and colour are some of the challenges when producing 3D printed biomedical products.

A newly discovered challenge when utilizing 3D printing technology in this study was the lifespan of materials. Respondent R3 mentioned that each material used has a limited life span and that this is an important factor to be considered. Theoretically, if a material is used after the specified expiry date, its properties might be affected, and this could lead to products that are harmful to the patient. From a clinical point of view, this could lead to failures such as excessive wear, fracture, or discoloration.

*“All the material(s) have (expiry) date(s). The lifetime for the resin is very short. Therefore, the expired material is very (challenging) for us. When we purchase a syringe of composite, three important aspects are the storage condition, batch number and the expiration date. Most of the direct materials have a limited shelf life.”* – Respondent 3

According to Respondent R3, all the materials have an expiry date; for example, the resin's lifetime is very short. Therefore, the selection of appropriate materials with a long-life span is important. Expired materials cannot enter the human body as it would then adversely affect the patient. This is due to the reduction in the product quality, which makes the product become brittle and causing cracking and discolouration. Hence, the research and development of novel resins with a longer lifespan must be intensively conducted to overcome this problem.

#### 5.1.2 Printers

Low dimensional accuracy is a challenge in the utilization of 3D printing technology to produce 3D printed biomedical products. The design plays an important role in producing highly accurate 3D printed products. Respondent R1 said that the accuracy of 3D printed biomedical products depends on the design. For example, variations in curing and cooling can lead to shrinkage or warping. Respondent R2 also supported the statement of Respondent R1 and mentioned that:

*“Long (and) thin unsupported features or a flat surface will cause low dimensional accuracy of a 3D printed product.”* –R2

Respondent R2 added that:

*“Accuracy also depends on materials. For instance, standard SLA resin will produce more dimensionally accurate parts than flexible resin. Standard materials are recommended for parts where high accuracy is critical”*– R2

From the transcribed data, R1 and R2 informed that the design and materials play important roles to produce biomedical products. According to R1 and R2, the design and materials play important roles in producing highly accurate 3D printed biomedical products.

Therefore, it can be concluded that developing the exact shape, size, and minute geometrical textures on artificial biomedical implants are essentially important for its proper functionality [97]. However, it is difficult to produce 3D printed biomedical products with the exact size and structure when using randomly selected machines and materials. Thus, if the dimensional accuracy is low, then the product will not fit in the body, and, at the same time will affect the clinical success rate of the product. Machines and materials should be carefully chosen to achieve the appropriate level of accuracy. According to Bertol *et al.* [98], the dimensional accuracy of the printed implants measured by 3D laser scanning showed an average of 200 μm, which allows its application in craniofacial structures [98]. Meanwhile, according to Osman *et al.* [99], digital light processing (DLP) has proved to be efficient for printing customized zirconia dental implants with sufficient dimensional accuracy. Hence, to produce 3D printed biomedical products, low dimensional accuracy is the main challenge. Therefore, the engineer and doctor should prudently choose the right machines and materials to produce 3D printed biomedical products.

In 3D printing technology, powder agglomeration is another challenge when producing 3D printed biomedical products. The binders are difficult to eliminate during sintering and this leads to poor densification when non-homogeneous microstructures result from agglomeration with larger pores.

According to Respondent R2:

*“It is difficult to produce (a) printed product (without) relating to the agglomeration of powder. Compared to other 3D printed products, 3D printed biomedical products have more problems related to the agglomeration of powder. The large pores caused by agglomeration can affect the 3D printed biomedical product and sintering temperature can affect the densification, behaviour, microstructure, and porosity of 3D printed biomedical products.”* –R2

In a nutshell, the limitation of powder agglomeration is one of the challenges in the utilization of 3D printing technology to produce biomedical products. Agglomeration can affect the process of producing 3D printed parts, such as causing low densification, which is very difficult to eliminate during sintering.

Therefore, the powder for 3D printing needs to fulfil certain requirements for the successful printing of 3D printed products. The required accuracy, such as in the layer thickness for z direction as well as print resolution for x and y directions defines the upper boundary for the particle sizes. Handling and processing properties, such as a tendency to agglomerate, electrostatic charging, and flowability that diminishes below a certain particle size should also be considered. Thus, if powder agglomeration occurs, the product will crack and produce large pores. Hence, the particle distribution needs to be carefully set to avoid powder agglomeration [90].

The printer nozzle size is another challenge in the utilization of 3D printing technology for biomedical products. The diameter of the nozzle directly affects the 3D printer extrusion width of each line in the product.

According to Respondent R1:

*“Now, in theory, smaller sizes of the nozzle(s) do allow (us) to achieve successful precision.”* –R1

Respondent R3 stated that:

*“If you use (a) 3D printer for doing large quantities of 3D printed biomedical products, you will want to make sure your extruder is laying down the right amount. Depending on the 3D printer, several nozzles can be interchanged reasonably easy.”* – R3

From the interviews, Respondent R3 supported the answers of R1 whereby the size of the nozzle can be considered a challenge in the utilization of 3D printing technology for biomedical products. The size of the nozzle is very important to ensure that the production of 3D printed biomedical products occurs smoothly. Smaller nozzle sizes can allow for the construction of biocompatible and biomimetic complex tissues.

According to Do *et al.* [100], the shear stress from the multi-sized nozzles could negatively impact cell viability during the printing process. Meanwhile, Patra *et al.* [101], stated that nozzle size will affect the viability of the materials printed. The nozzle size also affects the stacking of different printing paths. For example, the round nozzle could produce a product with a cross-section of an elliptical shape, and, hence result in high void density in the printed part. The nozzle size also affects the surface finish of the part because of the staircase effect, especially in large-scale 3D printing [102]. Conversely, Blaeser *et al.* [103], pointed out that the level of shear stress is directly influenced by different printing parameters, such as nozzle diameter. These phenomena are even more crucial in bioprinting, where hydrogels of high viscosity and small nozzles are applied to improve the final printing resolution. In conclusion, when selecting the 3D printing nozzle size, the major factor is all about balancing how much filament is extruded and the speed of the process. A smaller nozzle size allows the manufacturer to achieve better precision in printing.

A new challenge has been identified, which is to customize the fit and design of a 3D vascularized organ. For example, skulls have irregular shapes, and so it is difficult to make cranial implants. Implants and prostheses can be made in any imaginable geometry through the translation of X-ray, MRI, or CT scans into digital STL files. According to Respondent 1, the engineer must ensure that the fit and design of the object is customized to a desired shape, size and fit. A design is provided according to the size and specifications of a certain patient and it cannot be used for other patients because each human has unique body parts.

Respondent R1 stated that:

*“Because this is a patient's specific implant that I designed for you, so I cannot use this product for your friends. This is because the design just (fits) your body. So, if I design one for you, I cannot use that design for your friends.”* – R1

In order to bio-print thick tissues, highly repeatable and straightforward technologies and protocols should be developed in a logical manner, beginning from simple to difficult steps. For example, the eardrum is a very small part. Hence, it is very challenging for engineers to produce an eardrum of a certain size or specification according to a patient. Respondent R2 said:

*“Okay, I give the example, eardrum. So, get the test, limitation printed. This printer can go up to 5 microns.”* – R2

Respondents R1 and R2 stated that a product is designed according to the size and specification of certain patients and cannot be used for other patients. Therefore, customizing the fit and design is one of the challenges in producing 3D printed products. In conclusion, multi-physics, as well as analytical and computational modelling techniques should be used to determine the best microarchitecture for specific applications. All the relevant mechanical, biological, and physical properties of the biomaterial should be considered when producing a 3D printed object.

Furthermore, a new challenge in 3D printing technology in this study is the layer height. All 3D printing methods are based on a layer-by-layer building of a part. Printing is fast and produces the best prints with the right layer height. Choosing the appropriate layer height with the most accurate material setting is another challenge in utilizing 3D printing technology for biomedical products.

A high layer height usually results in a printed part with hard surfaces. The downside to this is an increase in the time to complete a print. Examples of processes, such as that used by FDM and SLA machines, prove that layer height is an important design parameter that impacts the printing time, cost, visual appearance and physical properties of a printed part. Respondent R2 mentioned that:

*“Printing parameters like layer height play a crucial role in fabricating biocompatible scaffolds with required mechanical strength and pore size. Layer height is ordinary. The faster it prints, the less the quality of the product. If we want to produce a delicate model and want to be 30 millimetres (mm), then we have to set it up for slow production. Because, a higher layer means lower quality.”* – R2

Respondent R2 believes that another challenge of utilizing 3D printing technology for biomedical products is the need to choose the right layer height with accurate material settings. This is because the faster the printer prints, the lower the quality of the biomedical product. To produce a delicate model, the set up must be for a slow production. A higher number of layers means that a lower quality product is produced.

In order to cope with this challenge, the engineer must optimise the best layer height by conducting numerous experiments to check and seek a solution. 3D printing builds a printed part by printing one layer at a time. Each subsequent layer is printed on the previous layer, and, finally, builds the desired 3D shape. Then, in order to make a solid and reliable final print, the engineer ensures that each layer is fully bonded to the layer below it. Furthermore, the engineer needs to make sure that the layer height matches the nozzle diameter.

Lastly, “build failure” is another new emerging challenge in 3D printing technology. The common cause of this is due to 3D materials that are not lying horizontal on the build plate when preparing the software, including rafts that cause the print to separate from the base, not adding supports when a model has any part overhanging in empty space, and creating models that are too thin. The “build failure” can also occur when the filament is jammed, or when there is loss of power or from extrusion errors.

According to Respondent R1:

*“To produce 3D printed biomedical products using the printer, the first step is to export the file from the computer to the printer. Next, the printer will process the information contained in the file and then will print the . This situation is called “build failed”.* –R1

Respondent R3 also said:

*“Sometimes, we do the production of (a) 3D printed biomedical products. The challenge we face is (when) the build failed. This happens when the machine (loses) power suddenly. So, it will look like “spaghetti”.* –R3

Based on the interview sessions, Respondent R3 supported the answer of R1, who said that “build failed” can be considered a challenge in the utilization of 3D printing technology for biomedical products. When this happens, the printing process should be restarted beginning with the first step. The best way to prevent over extrusion is to ensure that the layer height is less than the nozzle diameter and the speed of the cooling fan is increased. Additionally, to avoid this issue, the engineers should check the nozzle for clogs and increase the hot-end temperature [104].

### 5.2 Challenges in management

Possessing a high level of knowledge and skill in using software is very important for producing 3D printed objects. Company X provides training programmes for new employees in order to produce high quality products with high dimensional accuracy and features. Various programmes are conducted, such as mentor-mentee, employee exchange programmes to Belgium, and others to obtain new experience. As for the business or sales sector, employees will have access to taks or training for their workers.

Respondent R1 stated that:

“*We have training in Belgium for new workers, so new employees can get the new information about 3D printing technology. Even here, we have dedicated trainers. The trainers are (workers) who (have) been working a long time. So, those trainers will be mentors for (newbies). Therefore, usually we will set the programme or training for them and do it internally. If it is about business or sales, we will have access to another company to come here to do some training or talk. Usually because of many years of experiences (in) 3D printing, we have internal trainers that can give training or (talks).*”

Respondent R3 also supported Respondent R1, by stating that the re-education of staff can be considered as a challenge in the utilization of 3D printing technology. According to Respondent R3, their company is not just making normal 3D printers that are commonly available. It aims to make 3D printers to produce biomedical products with high precision. Therefore, employees working in this company must possess the expertise. The company provides training to its employees because it has several working procedures that need to be followed and it requires employees with expertise for these roles. All employees need to gain expertise in diverse physicochemical and biopharmaceutical characteristics of active pharmaceutical ingredients (APIs) through each stage of product development. Respondent R3 mentioned that:

“*We are not just making a normal 3D printer that is available on the Internet. We are aiming to make 3D printers that can print with high precision. The (workers) required to work in the company (have) to be (experts). So, the company (provides) some extra training for the (workers) so that they become (experts).*”

Respondents R1 and R3 implied that the re-education of staff can be considered a challenge when utilizing 3D printing technology. The employers of Respondents R1 and R3 are serious about upgrading their employee education and skill levels. In order to produce biomedical products, employees need special skills, like additional information pertaining to the biocompatibility of materials, the process of producing 3D printed biomedical products and how to design these biomedical products. This is because these companies must closely follow certain product or industry specifications.

The demands and expectations of 3D printing technology are high. Therefore, engineering and technical skills are required for the successful deployment of a wide range of 3D printing technology, from product design, material, technology, and, lastly, data management. At the same time, successful engineers must be creative, resourceful, and ready to “figure things out” in an industry that continues to develop and evolve. Therefore, the re-education of staff can be considered a challenge in the utilization of 3D printing technology for biomedical products.

Apart from that, the materials for 3D printing are very costly. The cost of buying a 3D printing machine is one of the most significant cost elements involved in utilizing 3D printing technology in the manufacturing industry. The price of a 3D printer is very expensive, ranging from 116,000 USD to 232,000 USD. The price of the machine depends on the ability of the machine to produce a product with certain specifications. A lower price means lower print quality, materials, build size, and functionality.

“*Second, the price of this machine is very expensive. To start the project, (USD)116 thousand to 1 million is required. But now, the bio-printer that we bring is affordable, below (USD)232 thousand. So, we have a goal (that) in Malaysia all universities (should) have a bio-printer.” – R2*

In conclusion, Respondents R2 and R3 agreed that the cost of machines is a challenge when utilizing 3D printing technology for biomedical products. The best 3D machines for the manufacturing industry are those that are reliable, easy to use and maintain, and that are capable of producing accurate and detailed prints. In addition, the 3D printing machine needs to be large enough for complex items and versatile enough to handle different materials. Conversely, the price of materials needed for producing 3D printed biomedical products is expensive because each biomaterial has specificic requirements in terms of material, mechanical and chemical properties, as well as cell-material interactions, processing methods, and the need for FDA approval [5]. Therefore, the cost of machines and materials used is a challenge when utilizing 3D printing technology for biomedical products.

Besides that, the size of the company does not affect the adoption of 3D printing technology for biomedical products. The assumption is that an increase in productivity is not due to the size of the organisation. According to Respondent 2:

*“(The) size of (the) company (does) not affect the (utilization) of 3D printing technology. This is because the company only needs some experts to produce biomedical products.”* –R2

In conclusion, the size of the company does not affect the adoption of 3D printing technology for biomedical products.

Next, the procedures and standards required for using 3D printing technology is also a challenge for the management of 3D printing technology companies. The procedures and standards required for using 3D printing technology is currently complicated. Each company also has its own standards when supplying medical products. For example, the medical products supplied to customers must be safe for human consumption. For industrial products, companies need to ensure that their product functions as per the requirements. Different products have different standards and uses different materials and processing methods. According to Respondent R2, the company also needs to apply for permission from the International Organization for Standardization (ISO) to invent and use 3D printing technology for producing biomedical products. According to Respondent R1:

*“When providing services to customers, certain standards must be followed. We have standards when supplying medical products to customers. For example, the medical product supplied to customers must be safe for the patient. For industrial products, we need to ensure (that the) product functions well. Different products have different standards as well as the material and processing method used (employed).”* – R1

Respondent R2 mentioned that:

*“Many procedures need to (be undertaken) such as (the) need to apply (for) permission from ISO. After (obtaining) the permission from the ISO, we (will then) continue to produce the products.”* –R2

Based on the collected data, two out of three respondents agreed that the procedures and standards are among the challenges in the utilization of 3D printing technology for manufacturing biomedical products.

Furthermore, cybersecurity is also a challenge in the management of 3D printing technology for biomedical products. According to Respondent R1, malicious cyber-attacks can affect the physical performance of 3D printing machines, the equipment, STL file and the component in the manufacturing system, which can cause a change in the shape, structural stiffness, natural frequency, and weight of the biomedical products. Respondent 3 also supported this statement when they said:

*“When we run the production using 3D printing technology, a malicious input could come from an integrated connection layer in the form of a malicious real-time controlling command that can change the production design.”* – R3

Hence, cybersecurity issue is another challenge to 3D printing technology used for manufacturing biomedical products. The sabotage can be executed remotely via internet access, which is ubiquitous in the 3D printing technology environment. The entire 3D printing technology data chain from design to manufacturing needs to be secured to maintain the integrity of both the digital data and the physical printed product when using 3D printing technology.

Marketing is also a new emerging challenge in the production of 3D printing technology. Data analysis shows that only two respondents implied that marketing is considered a challenge when utilizing 3D printing technology for producing biomedical products. They believe that, in Malaysia, the marketing of 3D printing technology for biomedical products is still in the infancy stage compared to Europe, the USA, or Singapore. In Europe and the USA, 3D printing is really in the mainstream and most of the medical divisions know about 3D printing. However, in Malaysia, there are not more than ten companies that apply 3D printing technology in their manufacturing businesses. Not surprisingly, not many Malaysians know of the existence of 3D printing technology in the production of biomedical products. This can be seen in the following statements:

*“Marketing is also another challenge, especially in the Asian market. This is because in Europe and the USA, 3D printing is really in mainstream use and most of the medical divisions know about 3D printing. "*– R1

Respondent R2 said that:

*“When we joined some events and conferences, some people were clueless about 3D printing because they (have) never heard of the technology. And it is possible that most people still (do) not know about the existence of 3D printing technology for manufacturing biomedical products in Malaysia.”* –R2

Simply put, Respondents R1 and R2 believed that the marketing of 3D printing technology for manufacturing biomedical products in Malaysia is still at the infancy stage compared to Europe, the USA, or even Singapore. This is because people in Malaysia are unfamiliar with the use of 3D printing technology for manufacturing biomedical products. Thus, marketing is one of the challenges when producing 3D printed biomedical products and selling them. Therefore, every company needs to draw up effective marketing strategies (promotions and advertisements), so that netizens are aware of the existence of 3D printing technology in Malaysia. There are no shortcuts in achieving the goal of the 3D printing industry through a proper marketing strategy. The management needs to be ready to invest a lot of time, patience, effort and finances towards this goal. When they pay attention to key elements of a good marketing strategy, it will be easier to develop an effective and logical plan that will lead to the successful adoption of 3D printing technology in the manufacturing industry.

Lastly, based on the transcribed data, another new challenge in utilizing 3D printing technology for manufacturing biomedical products is the patent and copyright issues. Patents protect 3D printed biomedical inventions such as new designs, processes, machines, or chemicals [105]. The central idea is that patents protect ideas, not just expressions of them. The main effect of patents is to give their holders the right to challenge any use of the invention by a third party. Meanwhile, a copyright is to protect the expression of ideas. Artistic works are generally considered as expressions of ideas; for example, books, songs, and computer programs [105]. The patent and copyright issue is one of the challenges that exists in all companies. Thus, if people were aware of the process to make the software or invention and copied it, it would be difficult to prove the original owner of the software or invention and that other people had copied it. A 3D printed biomedical product is designed using computer-aided drafting (CAD) software, which produces files that contain proprietary information. The theft or loss of these files could be disastrous to companies, potentially leading to digital sabotage or design theft.

According to Respondent R1:

*“Yes! We faced (it). But, it (is) (mostly) (due) to the software when to make 3D printed biomedical products likes a leg.* For example, *a skilled hacker penetrated one of the remote sites' firewall and stole the technical design files. “Look-alike” products were then released to the market at a cheaper price. When this situation occurs, first, you need (to) make a report to (the) IPA (Intellectual Property Academy) and (say), “Ok, this is my invention. This is my product and I should have ownership, all right?” So, (it is the same) for software. Usually, if I make (a) software and then you also see the process that I used to make (the) software and you copy it, it is really hard to prove that (it) is my creation and (another person copied) my invention. (It is so), especially for software, because in (the) whole process of 3D printing, the software is very important for us. This is because we will start using CT or MRI, then convert the data into a 3D model and use the software for creating new things. So, software is our focus for IPA. So, your question on how important the software is, well the answer is Yes. It is very important to us.”* –R1

Meanwhile, Respondent R2 mentioned that Malaysia is approximately three to five years behind in utilizing 3D printing technology, with many inventions having been already patented abroad. However, there are still innumerable opportunities for the company to patent its biomaterial products. For example in the case of a common biopolymer such as alginate, they cannot register any patents because other companies are very advanced and have already patented numerous products in this field.

Respondent R2 mentioned:

“*In terms of (patents), there are many (patents). Indeed, we (looked) at 2016–2017 abroad, many (inventions) (had) been (patented). In Malaysia, we are late, three years to five years only in 3D printing technology. There are still many more opportunities for us to patent our own biomaterials products. Like (these) (bio-cells) (while showing in glass bottles), they are proprietary or self-brewing, the alginate. We cannot make the patents because we are late.*”

Respondent R3 agreed with Respondents R1 and R2. In his company, the formulation of materials or the invention of new products is very important. Therefore, all formulations or inventions are protected by copyrights and patents. Conclusively, based on the collected data, all respondents alleged that patents and copyright issues are among the challenges of utilizing 3D printing technology for biomedical products. It is suggested that the government provide incentives or establish a subsidiary to reduce the burden of companies having to deal with patents and copyright issues.

**Figure 3:**
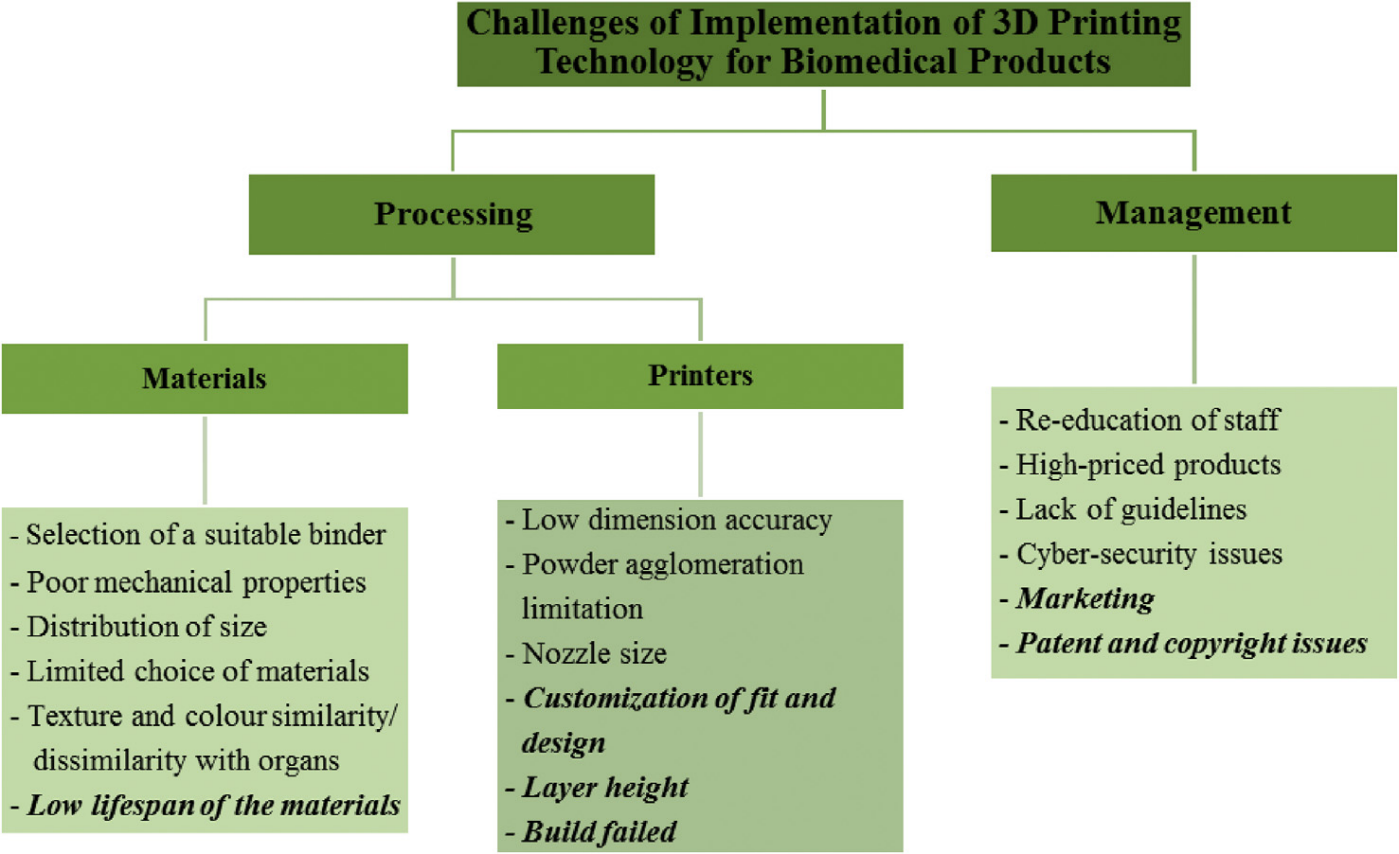
Overview of the challenges of 3D printing technology for biomedical products in Malaysia.

This study found several new elements in the challenge of utilizing 3D printing technology for manufacturing biomedical products. Figure 3 provides an overview of the challenges faced when utilizing 3D printing technology for biomedical products.

## 6 Conclusions

In summary, the results show that in respect of processing and materials, there are eight challenges when utilizing 3D printing technology for manufacturing biomedical products, which are as follows:

- *selection of a suitable binder*: various binders have varying effects on the product's biocompatibility, where the compatible one are the organic-based.
- *poor mechanical properties*: the product should have adequate tensile and compress strength also flexible rigidity after printing process.
- *low dimensional accuracy*: product fitting requires a precise design, the challenge is to overcome the shrinkage of the product during the curing and cooling process.
- *powder agglomeration limitations*: the particulate powder must be distributed evenly before sintering to prevent agglomeration and low densification product.
- *nozzle size*: appropriate nozzle size will determine the printed structure and design accuracy.
- *distribution of size*: over or under-fit particles may cause defects on the finished products.
- *limited choice of materials*: sources of raw materials for the construction of a similar and suitable product to human organs and tissues are still limited; and,
- *texture and colour similarity/dissimilarity with organs*: customer demands are always beyond current capabilities, so they need to be aware of limitations.

These challenges were faced by the core players of the existing industry in 3D printing technology for biomedical products in Malaysia, which then arises another four significant processing and materials challenges as follows;

- *low lifespan of the materials*: Inventory such as tracking records and storage of materials and product is crucial because most biomaterials have low lifespan, and expired compound reduces the quality of the product which makes the product brittle and causes cracking and discoloration.
- *customization of fit and design*: the concept of a product's recyclable design is difficult as the product is designed to the size and function of certain patients and can not be used in other patients.
- *layer height:* optimizing the best layer height is still dependent on multiple trials to check and find a solution that has been found as time consuming and costly.
- *build failed*: loss of connectivity or buggy control performance on software-hardware to perform tasks, resulting in failure of network and access to the set framework.

Apart from this, in the management aspect, there are four challenges when utilizing 3D printing technology for manufacturing biomedical products, which are re-education of staff, high-priced products, and lack of guidelines, and cyber-security issues. The size of the business is removed from the list of challenges because it was discovered that the size of a company or organization does not affect printing productivity. Nonetheless, marketing, patents, and copyright were found to be new challenges.

Overall, this study is important for the biomedical manufacturing sector as it offers information about the use of 3D printing technology for manufacturing biomedical products in developing countries such as Malaysia. This study could be a guideline for new manufacturers, human resources and the management sector. For new companies intending to adopt this technology, the qualitative sharing experience from this study will provide an early insight into what the company will encounter. It is anticipated that the findings of this study will assist Malaysians to obtain concise information about the utilization of 3D printing technology in the manufacturing industry.

Tackling the newbie's readiness to develop and implement this technology is critical, as is the confidence of the customers to purchase the products. This paper highlighted the fact that, to manufacture medical product, 3D printing technology is safe and effective. Hence, this paper hopes that the challenges discussed will encourage and empower newbies, policy makers, and government sectors to carefully adopt this technology and respond to consumer trust and demand appropriately.

## Declarations

### Author contribution statement

N. Shahrubudin: Conceived and designed the experiments; Performed the experiments; Analyzed and interpreted the data; Wrote the paper.

P. Koshy, J. Alipal, M. H. A Kadir: Analyzed and interpreted the data; Wrote the paper T. C. Lee: Conceived and designed the experiments; Analyzed and interpreted the data; Contributed reagents, materials, analysis tools or data; Wrote the paper.

### Funding statement

This work was supported by the Ministry of Higher Education and Universiti Tun Hussein Onn Malaysia for the financial support provided for this research through Research Grant Scheme, FRGS Vot K097 and Research Fund E15501, RMC UTHM.

### Competing interest statement

The authors declare no conflict of interest.

### Additional information

No additional information is available for this paper.
